# Genomic Sequencing of Ranaviruses Isolated from Turbot (*Scophthalmus maximus*) and Atlantic Cod (*Gadus morhua*)

**DOI:** 10.1128/genomeA.01393-16

**Published:** 2016-12-15

**Authors:** Ellen Ariel, Natalie K. Steckler, Kuttichantran Subramaniam, Niels J. Olesen, Thomas B. Waltzek

**Affiliations:** a College of Public Health, Medical and Veterinary Sciences, James Cook University, Townsville, Queensland, Australia; b Department of Infectious Diseases and Pathology, College of Veterinary Medicine, University of Florida, Gainesville, Florida, USA; c Technical University of Denmark, National Veterinary Institute, Frederiksberg C, Denmark

## Abstract

Ranaviruses have been isolated from Atlantic cod (*Gadus morhua*) and turbot (*Scophthalmus maximus*) in Denmark. Phylogenomic analyses revealed that these two ranaviruses are nearly identical and form a distinct clade at the base of the ranavirus tree branching off near other fish ranaviruses.

## GENOME ANNOUNCEMENT

Cod iridovirus (CoIV) is the earliest isolation of a ranavirus in fish. It was isolated from wild Atlantic cod in Danish coastal waters in 1979 during an investigation of ulcers in cod (1). However, results of infection trials could not definitively link the CoIV to the cod ulcerative disease (2). Ranavirus maximus (Rmax) was isolated from clinically healthy turbot fry from an aquaculture facility during a screening for export certification in 1999 (3).

The Rmax and CoIV isolates were propagated in bluegill fry (BF-2) cells as previously described (4) and harvested at third and fourth passages, respectively. Cell culture supernatant was clarified at 3,000 × *g* for 20 min, and total nucleic acids were purified using a DNeasy blood and tissue kit (Qiagen). The DNA libraries were prepared using the Nextera XT DNA kit (Illumina), and sequencing was performed using a V3 chemistry 600-cycle kit on a MiSeq platform (Illumina). *De novo* assembly of the paired-end reads in SPAdes (5) produced contiguous consensus sequences of 115,510 bp with a G+C content of 55.19% and 114,865 bp with a G+C content of 56.99% for Rmax and CoIV, respectively.

The genomes of Rmax and CoIV were annotated using GATU (6) with epizootic hematopoietic necrosis virus (EHNV, GenBank accession no. NC_028461) as the reference genome (7). Additional putative open reading frames (ORFs) were identified using GenemarkS (8), and gene functions were predicted based on BLASTp searches against the NCBI GenBank nonredundant (NR) protein sequence database. A total of 100 putative ORFs were predicted in Rmax and 98 in CoIV; compared to other closely related fish ranaviruses, 100 putative ORFs were predicted in EHNV and 111 in short-finned eel ranavirus (SERV, GenBank accession no. KX353311) (4). Comparative genomic analyses revealed that Rmax and CoIV are nearly identical to each other, except for a nonsense mutation leading to an early stop codon in CoIV (ORF70 in Rmax, unannotated ORF located between ORFs 66 and 67 in EHNV) and the absence of a gene in CoIV (ORF62 in Rmax, ORF58L in EHNV). An analysis of locally collinear blocks in Mauve (9) revealed that the genomes of Rmax and CoIV are collinear with each other and nearly collinear to EHNV and SERV, with the exception of a single minor inversion involving ORFs 95 and 96 in CoIV and ORFs 97 and 98 in Rmax (ORFs 95R and 96L in EHNV, ORFs 108 and 109 in SERV). Maximum likelihood phylogenetic analyses based on the concatenated nucleotide sequences of the 26 *Iridoviridae* core genes (10) revealed that Rmax and CoIV form a distinct clade at the base of the ranavirus tree branching near other fish ranaviruses (e.g., EHNV, SERV).

The isolation of nearly identical ranavirus strains from two different marine species, hundreds of kilometers and 20 years apart, indicates the persistence of a ranavirus strain of unknown virulence in the marine environment. With the exception of the grouper iridoviruses in Asia (11), the impact of ranaviruses on mariculture remains unclear. Experimental challenge studies are needed to determine whether CoIV or Rmax pose a risk to marine fisheries.

### Accession number(s).

The complete genome sequences of Rmax and CoIV have been deposited in GenBank under the accession numbers KX574343 and KX574342, respectively.
